# Treating Deep Venous Thrombosis in a Background of Crohn’s Disease: A Clinical Conundrum

**DOI:** 10.7759/cureus.49831

**Published:** 2023-12-02

**Authors:** Gurinder Singh, Keerthana Panchagnula, Paavan Desai, Dhruvish Mistry, Aakash Baskar, Ashima Gupta, Kamya Mehta, Yashash Pathak

**Affiliations:** 1 Internal Medicine, Universidad Latina De Panama, Panama City, PAN; 2 Internal Medicine, Kasturba Medical College, Manipal, Bengaluru, IND; 3 Internal Medicine, Gujarat Adani Institute of Medical Sciences, Bhuj, IND; 4 Internal Medicine, K.A.P Viswanatham Government Medical College, Tiruchirappalli, IND; 5 Internal Medicine, Dr. Panjabrao Alias Bhausaheb Deshmukh Memorial Medical College, Amravati, IND; 6 Internal Medicine, Government Medical College Akola, Akola, IND; 7 Internal Medicine, Baylor Saint Luke's Medical Center, Houston, USA

**Keywords:** upper limb thrombosis, hemato oncology, peripherally inserted central catheter (picc), crohn’s disease (cd), deep vein thrombosis (dvt)

## Abstract

Deep venous thrombosis (DVT) commonly affects the lower extremities, often as a result of prolonged immobilization. However, upper limb DVT is an atypical presentation, typically associated with risk factors such as the use of a peripherally inserted central catheter (PICC) line.

This case report describes an uncommon case of DVT management in a patient with Crohn’s disease, a condition more frequently characterized by painful lower gastrointestinal symptoms and chronic diarrhea. A 22-year-old male with a history of Crohn’s disease developed swelling and purplish discoloration at the brachial site of a PICC line site. Laboratory results indicated anemia with a hemoglobin level of 9.9 g/dL and a hematocrit of 31.9%. Doppler ultrasound confirmed the DVT in the left long axillary, left subclavian, and left long basilic veins. Given the patient's concurrent lower gastrointestinal bleeding, a cautious approach was required to balance the risks and benefits of anticoagulation. Upon recommendation by Hematology, a prophylactic dose of enoxaparin was initiated and subsequently escalated to a therapeutic dose as tolerated. The patient's condition was closely monitored, and he successfully reached the full therapeutic regimen without complications. This case underscores the importance of individualized DVT treatment strategies in the context of concurrent Crohn’s disease, offering insights into managing anticoagulation in the presence of bleeding risks.

## Introduction

Deep venous thrombosis (DVT) is characterized by the formation of blood clots in the body's deep veins, and it emerges from a complex interplay of risk factors including prolonged immobilization, surgical history, intravascular devices, and certain chronic medical conditions such as Crohn's disease [1].

Crohn's disease is a chronic inflammatory condition that affects any part of the gastrointestinal tract from the mouth to the anus, with symptoms that include chronic diarrhea, lower gastrointestinal bleeding, and crampy abdominal pain typically localized in the right lower quadrant. The palpable mass may also be detected in the right lower quadrant [2]. Malabsorption is another significant concern; patients with small bowel involvement may experience watery diarrhea and steatorrhea, leading to protein-calorie malnutrition, hypocalcemia, vitamin deficiencies (e.g., vitamin B12), and metabolic bone disease. Additionally, the disease can lead to severe complications such as significant gastrointestinal bleeding and the development of enterocutaneous or perianal fistulas [3]. Intravenous therapies, often necessitated by Crohn's disease for long-term treatment, typically involve the use of peripherally inserted central catheters (PICCs). While PICC lines facilitate essential medication delivery and nutritional support, they also elevate the risk of DVT, adding a layer of complexity to patient care [4].

Patients with Crohn’s disease who require PICC lines face a compounded risk for DVT. Anticoagulation therapy, the cornerstone treatment for DVT, introduces the potential for iatrogenic bleeding [5]. This is a notable concern in Crohn's disease where lower gastrointestinal bleeding (LGIB) is a prevalent issue [6]. The confluence of Crohn's disease, catheter-associated thrombosis, and the ensuing need for anticoagulation necessitates a meticulous risk-benefit analysis, often requiring specialist consultation to navigate the intricacies of such a dual diagnosis.

The intersection of Crohn's disease and DVT, particularly against the backdrop of PICC line utilization, remains a challenging domain with limited consensus in the literature, underscoring the imperative for individualized care plans. This case report delves into the intricacies of managing a patient at this intersection, illustrating the need for astute clinical judgment and an integrative multidisciplinary approach. We present a detailed complex case analysis, drawing attention to the critical decision-making process and the strategies employed to optimize patient care while judiciously mitigating risk.

## Case presentation

**Figure 1:**
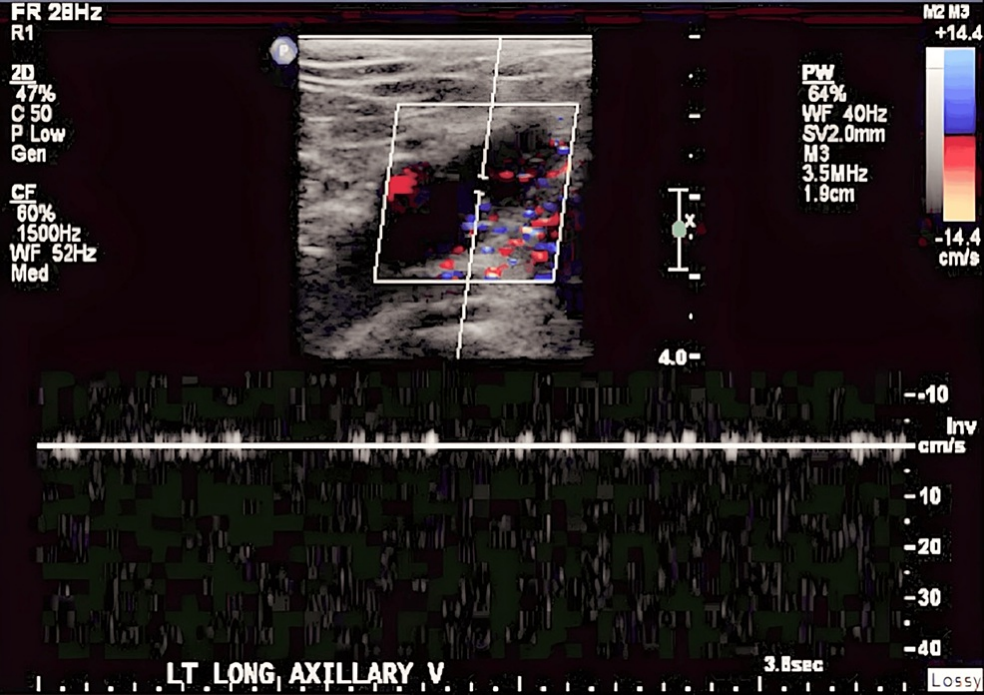
Venous Doppler of left long axillary vein showing thrombosis

**Figure 2:**
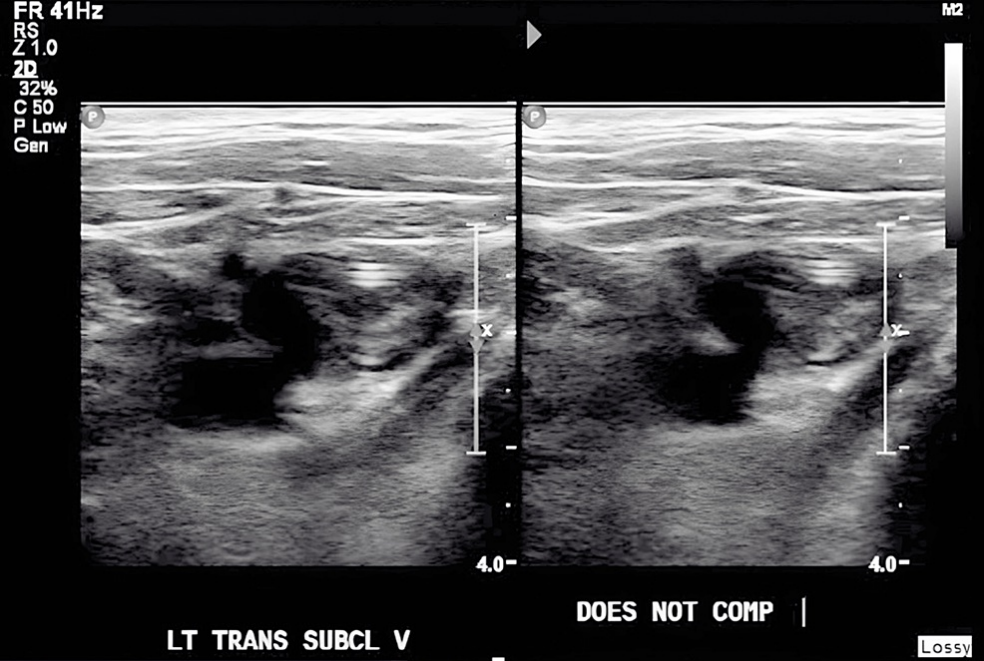
Venous Doppler showing thrombosis in the left subclavian vein

**Figure 3:**
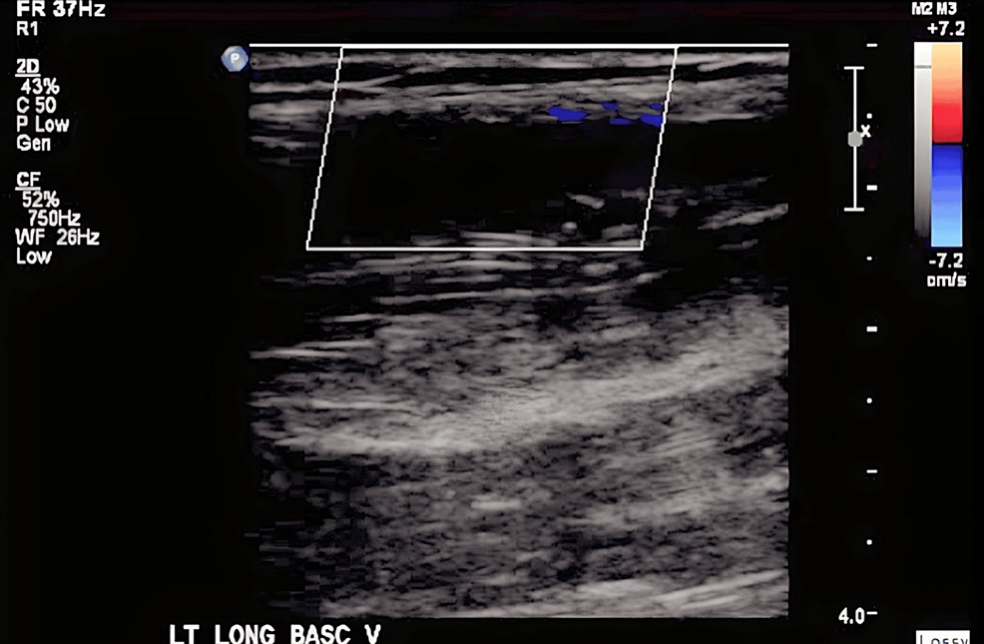
Venous Doppler showing thrombosis in the left long basilic vein

We present the case of a 22-year-old male patient with a past medical history of autism spectrum disorder and ulcerative colitis, The patient underwent total colectomy followed by end ileostomy. Later, he had a completion proctectomy with J-pouch creation and diverting loop ileostomy. A year after, an ileostomy reversal was performed. However, he experienced several complications, including haematochezia, anastomotic ulcers, cuffitis, and perianal abscesses. He was admitted for sepsis, secondary to perirectal abscess status post-seton drain placement and pouchoscopy. Pouchoscopy revealed early-onset pre-pouch ulceration, cuffitis, and severe gastrointestinal bleeding due to recurrent pouchitis consistent with Crohn's disease. The patient was treated with IV steroids and antibiotics and was started on total parenteral nutrition via a left upper extremity (LUE) PICC for severe protein-calorie malnutrition before being discharged. A venous Doppler of the left upper extremity was ordered due to clinical observations of swelling and purplish discoloration and revealed acute thrombosis in the subclavian and axillary veins of the left deep venous system (Figures 1, 2) and acute thrombus in the basilic vein of the left superficial venous system (Figure 3).

The patient returned due to acute swelling at the LUE brachial PICC site, which his mother had noticed the previous night. The swelling extended from mid-bicep to elbow, associated with purplish discoloration. The patient denied experiencing pain, numbness, weakness, or other changes in sensation. He exhibited a full range of motion in all extremities and denied fever, chills, chest pain, or shortness of breath. There was no discharge observed from the PICC site, and the patient was able to tolerate both oral fluids and foods. Upon physical examination, vital signs were within normal limits; however, lab results indicated a decrease in hemoglobin levels to 9.9 g/dL and a reduction in hematocrit to 31.9%.

In summary, our patient with a complicated medical history and severe malnutrition developed acute LUE brachial PICC site swelling associated with acute venous thrombosis. Appropriate measures were taken to manage the thrombosis and mitigate potential complications.

Treatment plan

The treatment plan for the patient included several actions to address the medical condition. Firstly, anticoagulation therapy was initiated with enoxaparin 60 mg bis in die (BID) was prescribed for three months to treat acute venous thrombosis. Additionally, the PICC line was removed from the affected extremity to prevent further complications. Before the commencement of the anticoagulation treatment, it was crucial to ensure there was no active LGIB. Considering the elevated risk of bleeding, a consultation with Hematology was arranged. Their recommendation was to start the patient on a prophylactic dose of enoxaparin and then progressively increase to the full dose, contingent on the patient's tolerance. The healthcare team also considered alternative means of nutrition delivery, such as central venous catheter insertion in a different site or enteral nutrition.

For the management of Crohn's disease, the patient continued with oral steroids (prednisone 30 mg then weekly taper by 5 mg) and mesalamine 1000 mg suppository. However, the patient did not take the suppository citing discomfort as the reason. As part of the ongoing monitoring daily checks of his hemoglobin levels were instituted. To ensure early detection of any potential complications, close monitoring for lower gastrointestinal (GI) bleeding and hematochezia was also conducted. Furthermore, a nutrition consult was scheduled to evaluate the patient's dietary needs.

The patient's sodium levels were monitored, and oral fluid intake was encouraged. The patient continued taking other prescribed medications, such as famotidine 20 mg tablet, ferrous sulfate 325 mg tablet, lactobacillus rhamnosus, GG, 10 billion cell capsule, potassium, sodium phosphates 280-160-250 mg powder packet, tramadoL 50 mg tablet, multivitamin, thiamine, and folic acid supplementation.

Expected outcome of the treatment plan

The primary objective of the plan was to enhance the patient's nutritional status and overall well-being. Concurrently, the plan aimed to manage symptoms associated with Crohn's disease while thwarting potential complications. By achieving these outcomes, the healthcare team aspired to elevate the patient's quality of life and pave the way for successful recovery.

Actual outcome

The patient was admitted for anticoagulation optimization and PICC replacement. After initiating anticoagulation therapy with Lovenox, the patient's LUE edema and related symptoms were managed. The patient's nutritional status was addressed through a nutrition consult, continuing home supplements, and encouraging oral intake. The patient's anemia was managed with oral iron supplementation, and his sodium levels were monitored with encouragement of oral fluid intake. Consultations with GI and surgical teams were conducted for further management of the patient's Crohn's disease and surgical complications.

## Discussion

Secondary DVT of the upper limb occurs due to thrombosis surrounding the indwelling central venous catheter, as illustrated in the discussed case [7]. Initial treatment involves IV heparin, followed by direct oral anticoagulants (DOAC). If the central venous catheter is no longer needed, it should be removed. Alternatively, a new one can be placed in the opposite arm. Thrombolysis is another treatment option for more proximal upper extremity (UE) DVTs. However, this method is contraindicated in patients with a high risk of bleeding [8].

Patients with inflammatory bowel disease (IBD) face an increased risk of bleeding when they are put on anticoagulants (ACs) like heparin or DOACs. The incidence of major bleeding in such patients was found to be 2.6/100 patient-years during the periods of exposure to ACs, and 0.9/100 patient-years during unexposed periods [9]. In the aforementioned case, the patient's history of Crohn's disease complicated DVT treatment. Nevertheless, given the gravity of potential complications like pulmonary embolism, the imperative for anticoagulation outweighed the risk of a potentially severe LGIB [1].

Had the DVT been located in the lower extremity, the situation would have had an indication for the placement of an inferior vena cava filter [10]. Since no equivalent alternative exists for UE DVTs, close monitoring was the chosen strategy. Fortunately, our patient experienced rapid LGIB resolution, eliminating the need for treatment alteration. Should severe LGIB have arisen, catheter-directed thrombolysis would have been the favored alternative, as it poses a lesser bleeding risk compared to systemic thrombolysis [8].

## Conclusions

DVT in patients with underlying conditions, such as Crohn's disease, poses unique clinical challenges, especially when presented alongside complications like LGIB. The simultaneous presence of a PICC line further amplifies these challenges. In the presented case, the need to balance the urgent requirement of anticoagulation for DVT management against the risks associated with exacerbating LGIB due to Crohn's disease necessitated a judicious, individualized treatment approach.This case underscores the critical role of multidisciplinary collaboration in the care of complex patients. It highlights the importance of continually assessing the risk-benefit ratio for each treatment strategy in dynamic clinical scenarios. While the treatment was successful in this particular case, it brings forth a broader discussion on the best practices in similar clinical situations. It serves as a compelling testament to the challenges faced in the management of chronic inflammatory diseases and reiterates the significance of individualized care to optimize outcomes while minimizing risks.
